# Nasal Planum Vasculopathy in a Scottish Terrier Dog Treated with Ciclosporin and Endonasal Stents

**DOI:** 10.3390/vetsci5030073

**Published:** 2018-08-15

**Authors:** Roberta Sartori, Valeria Colombo, Silvia Colombo, Chiara Noli

**Affiliations:** 1 Servizi Dermatologici Veterinari, Milano, Italy; 2 Ambulatorio Veterinari Associati Castelnuovo e Colombo, Magenta (MI), Italy; vale.colombo64@alice.it; 3 Servizi Dermatologici Veterinari, Legnano (MI), Italy; colombo_silvia@yahoo.it; 4 Servizi Dermatologici Veterinari, Cuneo and BiEsseA Laboratorio Analisi Veterinarie, Milano, Italy; pitnoli@iol.it

**Keywords:** vasculopathy, nasal planum, Scottish Terrier, ciclosporin, endonasal stents

## Abstract

A two-year-old, intact female Scottish Terrier presented with one-and-a-half-year history of erosive and ulcerative lesions affecting the nasal planum. Clinical appearance, history, histopathology, and response to therapy were suggestive of a rare vasculopathy of the nasal planum that has been previously described in Scottish Terrier dogs. In previously published reports, medical treatments of the disease had failed, leading to euthanasia of five dogs, while a short-term follow-up was available for one case that was controlled with prednisolone and ciclosporin. The dog reported herein was successfully treated with medical therapy consisting initially of a combination of ciclosporin and prednisolone and endonasal stents applied over the first six months. Stents were inserted in order to prevent abnormal scarring and nostril stenosis. More than one and a half years after diagnosis, the dog is still being administered ciclosporin once daily, breathes normally, and has an optimal quality of life.

## 1. Introduction

Familial vasculopathy of the nasal planum is a very rare disease reported in Scottish Terrier dogs [1]. In 1991, five cases were described in related puppies in Denmark [2]. In these cases, antibiotic, antifungal, and immunosuppressive therapies were unsuccessful, and all dogs were euthanized. Two new cases from Argentina and United States were described in 2009 [3]. These cases were treated with a combination of ciclosporin and prednisolone. One of them was lost to follow-up, while in the second case, ciclosporin arrested the progression of the disease and a short-term follow-up was available. To the authors’ knowledge, no more cases have been published to date. The case reported in this paper is the first time the disease has been successfully treated with both medical therapy and endonasal stents.

## 2. Case Presentation 

**Figure 1:**
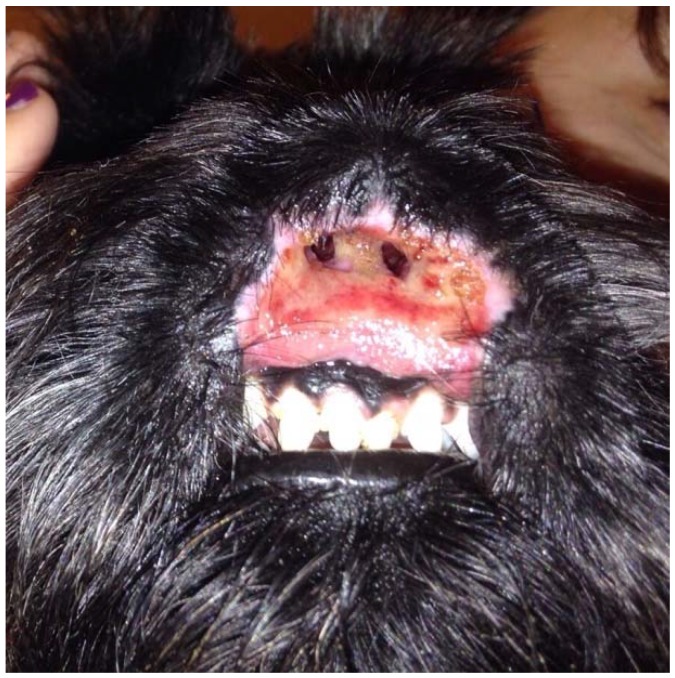
Wide and deep ulcer destructing and replacing the nasal planum, philtrum, and upper lip.

A two-year-old, intact female Scottish Terrier dog presented with erosive and ulcerative lesions affecting the nasal planum (Figure 1). These lesions were neither painful nor pruritic and had been present since the dog was four months old. There was no familial history of a similar condition affecting the parents or the littermates.

**Figure 2:**
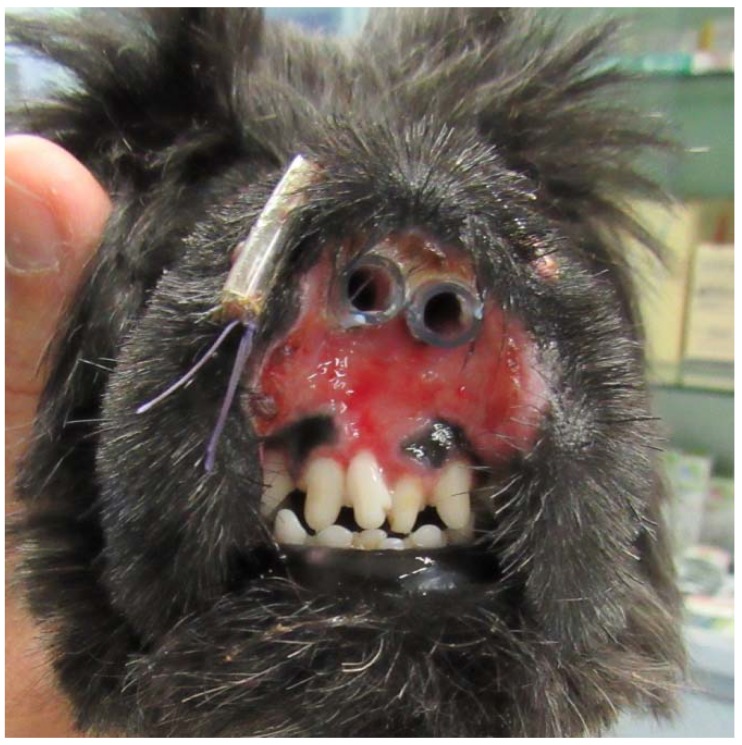
Frontal view of the muzzle after the application of endonasal stents.

The dog had initially been treated by the referring veterinarian with short courses of amoxicillin-clavulanic acid (Synulox; Zoetis, Rome, Italy) 25 mg/kg orally twice daily combined with prednisone (Deltacortene; Bruno Farmaceutici, Rome, Italy) 0.5 mg/kg orally once daily, leading to a temporary improvement, followed by progressive worsening of the condition. *Pseudomonas aeruginosa*, which was sensitive to gentamycin and quinolones, was isolated from the nasal tissues by a bacterial culture performed by the referring veterinarian when the dog was nine months old. Marbofloxacin (Aristos; ATI, Ozzano nell’Emilia, Italy) 2 mg/kg orally once daily was administered for one month without improvement. The referring veterinarian then administered methylprednisolone acetate (DepoMedrol Vet; Zoetis, Rome, Italy) 1 mg/kg intramuscularly, leading to clinical improvement of the lesions and abnormal scarring and stenosis of the nostrils. Three weeks later, erosions and ulcerations were progressively worsening, and the dog was referred for dermatological consultation. The dog was regularly vaccinated, dewormed, on heartworm prevention, and fed a commercial dry food. A fipronil-S-methoprene spot-on product (Frontline Combo; Merial, Milano, Italy) was applied monthly for flea and tick prevention. The patient had no history of previous diseases. On general examination, the dog appeared to be in good health. The rectal temperature was normal and respiratory and heart rates were within normal limits. Lymph nodes were normal in size. On dermatological examination, wide and deep ulcers were apparent, with complete destruction of the nasal planum, philtrum, and nostrils as well as the central part of the upper lips. The ulcerative process also affected the gum, in correspondence with the upper central incisors. These lesions were neither painful nor pruritic, and the dog could eat normally (Figure 1). Differential diagnoses considered for this presentation included nasal planum vasculopathy of the Scottish Terrier dog, dermal arteritis of the nasal philtrum, discoid lupus erythematosus, pemphigus complex, squamous cell carcinoma, and leishmaniosis. The latter was unlikely because the dog lived in a nonendemic area in Northern Italy and Immunofluorescence Antibody Test titer was negative. Cytological examination of cutaneous lesions revealed sparse cocci, rare neutrophils, and many erythrocytes and was therefore considered inconclusive. Biopsy specimens were collected by the referring veterinarian under general anesthesia with a 5 mm biopsy punch from erosions and ulcers. Histological examination of haematoxylin and eosin-stained sections revealed an irregularly hyperplastic epidermis, erosions, ulcerations, and granulation tissue, and it was not diagnostic. Even if the primary pathognomonic lesions were not observed, the clinical presentation and response to therapy were consistent with the diagnosis of a dermatosis previously described as vasculopathy of the nasal planum of the Scottish Terrier dog. The dog was treated with oral prednisolone, 1 mg/kg once daily (Vetsolone; Bayer, Milano, Italy) and oral ciclosporin, 5 mg/kg once daily (Atoplus; Elanco, Sesto Fiorentino, Italy) for three weeks. Prednisolone was slowly tapered and discontinued after 20 weeks of therapy, while ciclosporin was continued at the same dosage. Two stents were placed in the nostrils in order to prevent abnormal scarring and subsequent stenosis (Figure 2). Stents consisted of two sections of silicone drainage tubes measuring 1.5 cm in length and 0.4 cm diameter. These were inserted in the nares rostrally and fixed to the dorsolateral walls of the nasal cavities with a monofilament EP 3.5 suture material composed of polyglycolide-poly (e-caprolactone) copolymer (Monofil; Assut Europe, Rome, Italy) under general anesthesia. Stents were well tolerated by the dog and were removed and replaced twice, every two months, due to dissolution of the suture material. Stents were definitely removed after six months from the first application.

**Figure 3:**
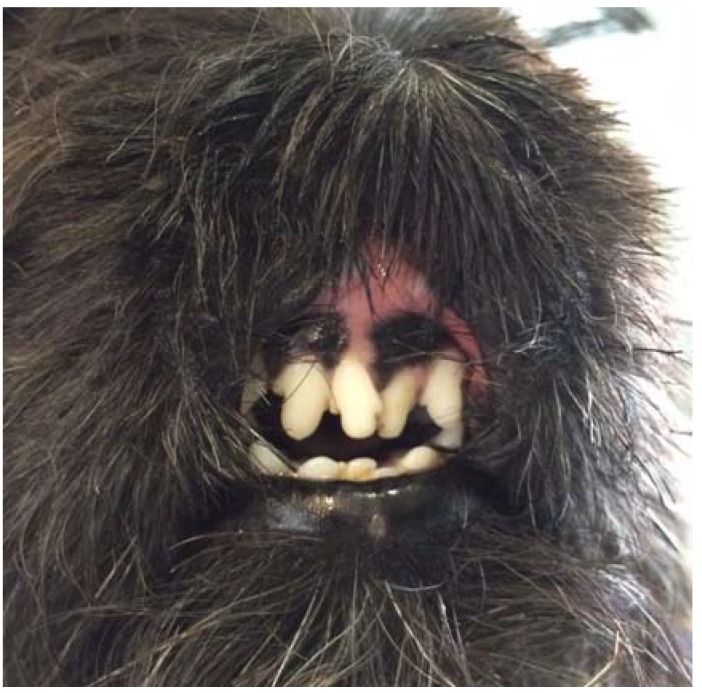
Frontal view of the muzzle five months after the removal of stents.

During the treatment, no adverse effects were observed except for polyuria and mild polydipsia. Haematological and biochemical profiles were performed once monthly. No relevant haematologic abnormalities were recorded. Biochemistry panel showed moderate elevation of alkaline phosphatase (ALP) consistent with glucocorticoid administration. All these abnormalities resolved when prednisolone was discontinued. During the first month of therapy, the dog’s condition showed improvement at the weekly rechecks. Ulcers, although still present, markedly decreased in size and depth. Erosive and ulcerative lesions completely regressed within five months after stents removal (Figure 3). There was complete re-epithelialization of the muzzle, although with severe scarring, and regrowth of hair at the periphery of the affected area. The nasal planum and the central part of the upper lip were absent, and two remnants of the nares were present; however, the dog could breathe and eat normally. More than one and a half years after the start of the medications, the dog is still on treatment with 5 mg/kg ciclosporin orally once daily, breathes and eats normally, and the quality of life is excellent.

## 3. Discussion

A familial vasculopathy of the nasal planum was initially described in 1991 in five related Scottish Terrier puppies, and only two further cases were reported in 2009 [2,3]. The condition was called “idiopathic pyogranulomatous inflammation and leukocytoclastic vasculitis of the nasal planum” in the initial report, while in the second one, the name “hereditary pyogranuloma and vasculitis of the nasal plane” was used. In previous case series, nomenclature of this disease clearly reflected histopathological presentation characterized by a pyogranulomatous inflammatory process and leukocytoclastic vasculitis. Histopathology of the case described herein showed necrosis, ulcers, fibrosis of the dermis, and vascular proliferation compatible to what had been previously described. Although the etiopathogenesis of this syndrome is unknown, the breed predisposition and the occurrence in related dogs with signs appearing by three weeks to one month of age strongly suggests a hereditary basis. Furthermore, the fact that 50 per cent of puppies of two litters were affected suggested an autosomal dominant inheritance pattern. However, this could not be demonstrated because breeding trials were not conducted [2]. An immune-mediated pathogenesis has been suspected^3^ because of the good response to high doses of glucocorticoids and the histopathological features of sterile pyogranulomatous inflammation and leukocytoclastic vasculitis. The onset of the disease at four months of age in this case may be consistent with genodermatosis, although the littermates were not affected. In the cases previously described, mucopurulent nasal discharge together with erosions and ulcerations of the nasal mucosa were initially observed. As the condition progressed, ulcers tended to enlarge and coalesce to involve the whole nasal planum, eventually leading to loss of the rostral nasal cartilages and central upper lip. In none of these cases, stenosis of the nostrils was described as it was observed in the present case. However, scarring and depigmentation were prominent [3]. Prognosis of the disease is usually guarded to poor. Cases reported in Denmark were all euthanized, with immunosuppressive doses of prednisone (2 mg/kg orally once daily) leading to temporary resolution of the lesions in one case [2]. The two dogs reported from North and South America were successfully treated with a combination of prednisolone and ciclosporin; however, only a short follow-up is available for one of them. The patient described in this case report was successfully treated with a combination of prednisolone and ciclosporin. Furthermore, due to prominent nares stenosis and impaired respiration, two silicone stents were inserted in order to prevent occlusion of the nostrils due to scarring. The result was optimal, and the stents could be definitely removed after six months. This is the first description of endonasal silicone stent application for this purpose. In veterinary medicine, metallic stents may be used in cases of nasopharyngeal stenosis or choanal atresia [4]. Stent placement has been used with success, but complications such as tissue in-growth, chronic infections, and the development of an oronasal fistula must be considered [4]. The case described herein did not develop any complication due to silicone stent placement, and the stents were well tolerated.

## 4. Conclusions

In this case, long-term control of the disease was achieved with both systemic immunosuppressive therapy and endonasal stents. Due to the suggested hereditary basis of this condition, the dog was neutered. Exclusion from breeding was suggested to all dogs from the same litter and to their parents.
